# A resident-led clinic that promotes the health of refugee women through advocacy and partnership

**Published:** 2019-11-28

**Authors:** Jocelyn Stairs, Navpreet Bal, Finlay Maguire, Heather Scott

**Affiliations:** 1 Department of Obstetrics and Gynaecology, Dalhousie University, Nova Scotia, Canada; 2 Faculty of Computer Science, Dalhousie University, Nova Scotia, Canada

## Implication Statement

Longitudinal global health experiences promote cultural competency and a commitment to caring for underserved populations beyond residency. We describe a longitudinal, co-curricular local global health experience. Obstetrics and gynaecology residents partnered with the Family Medicine-led Halifax Newcomer Health Clinic to provide education and clinical well-woman care to refugee women. This resident-led initiative meets the care needs of an underserved population while promoting resident engagement in health advocacy and inter-specialty collaboration.

## Déclaration des répercussions

Les expériences longitudinales en matière de santé globale favorisent la compétence culturelle et un engagement à prendre soin des populations mal desservies au-delà de la résidence. Nous décrivons une expérience longitudinale parallèle locale ensanté globale. Les résidents en obstétrique et gynécologie s’associent à la clinique de santé pour nouveaux venus d’Halifax dirigée par un groupe de médecine familiale pour offrir une éducation et des soins de santé préventifs aux réfugiées. Cette initiative géréepar des résidents satisfait les besoins en soins de santé d’une population mal desservie tout en encourageantla participation des résidents dans la promotion de la collaboration entre spécialités.

## Introduction

Canadian residency curricula must incorporate the health advocate and collaborator CanMEDS roles.^1^ As we move towards competency-based medical education, clinical opportunities to build skills in these areas will be essential to meeting competency in these roles. Evidence suggests that working with underserved populations during residency promotes cultural competency and leads to a commitment to serve these populations after residency.^2^ Residency programs are increasingly focused on the longitudinal integration of these global health experiences to foster a global consciousness.^3^^-^^5^

We describe a resident-led, co-curricular collaboration that provides a longitudinal local global health experience to obstetrics and gynaecology (Ob/Gyn) residents in collaboration with Family Medicine. We obtained IWK Research Ethics Board approval for the retrospective review of our clinic (No. 1024122).

## A collaborative partnership

Since 2015, Canada has welcomed 44,560 Syrian refugees.^6^ This has increased the number patients at Refugee Health Clinics.^7^ The Canadian Clinical Guidelines for Immigrants and Refugees identified cervical cancer screening and contraception as priorities for newly resettled refugees.^8^ This guideline identifies the creation of safe spaces for education and provision of clinical care as a method of improving uptake of these preventative health services.^8^

In October 2015, Ob/Gyn residents at Dalhousie University partnered with Family Physicians at the Halifax Newcomer Health Clinic (HNHC) in Halifax, Nova Scotia, Canada to run monthly well-woman clinics whose goals are to: educate refugee women about cervical cancer screening to promote uptake; provide contraceptive counselling and Intrauterine device (IUD) placement; create a discrete, female-centric environment; foster resident advocacy and cultural competency through longitudinal collaboration.

We initiated this partnership when Family Physicians at the HNHC approached residents in the Department of Obstetrics and Gynaecology to help with providing timely appointments during the initial influx of refugees and demand for IUD placements at the clinic, as they did not have a provider with the skills to provide this service. Following a successful pilot, we identified this clinic as an opportunity for ongoing partnership.

The Well-Woman clinic offers appointments to female patients who are seen through the HNHC. The clinic assigns a dedicated interpreter to women for the duration of their visit and gives them opportunity to participate in a resident-led teaching session prior to their appointment. Community partners and HNHC Physicians assist residents in identifying relevant education topics. Residents coordinate the clinic dates and resident volunteers. HNHC physicians supervise residents to provide pap testing, IUD placements and contraceptive counselling. An off-site gynaecologist provides additional supervision for more complex cases, if required. Please see Table 1 for a review of services provided. Fifty-four percent of Ob/Gyn residents in the current cohort have participated in this voluntary, co-curricular activity. Family Medicine residents, both male and female, may also participate in the clinic. One has volunteered thus far. Male residents have led teaching sessions, and this has been well received by patients. Patients choose to see a male or female resident for their clinical appointment.

**Table 1:** Newcomer Well-Woman clinic service provision October 2015-October 2018

		N (%)
**Number of Patients Seen**	2015	17
	2016	34
	2017	30
	2018	50
	**Total**	**131**
**Language Spoken**	Arabic	107 (79%)
	Amharic	7 (5%)
	Other*	17 (16%)
**Pap**	Yes	85 (65%)
	No	46 (35%)
**IUD**	Copper	8 (6%)
	Levonorgestrel IUS	9 (7%)
	**Total**	**17 (13%)**

* Other includes Dari, English, Farsi, French, Nepali, Spanish, Swahili, Tigrinya

Residents noted increased interest in copper IUDs in this population. As these are not currently covered for government assisted refugees under the Interim Federal Health Program, residents have partnered with a local advocacy group to write a letter to the Nova Scotia Minister of Health to recommend that they be added to the provincial formulary to secure coverage.

## Future directions

The Department of Obstetrics and Gynaecology is exploring the addition of an onsite gynaecologist to supervise residents in providing a gynaecology consult clinic at the HNHC site and the integration of this clinic into the formal curriculum. This would complement the Department’s existing didactic Global Health curriculum. Educators identify this combination of didactic and clinical curricula as integral to a comprehensive training program in global health.^5^ Additionally, we are currently preparing a Research Ethics submission to seek patient feedback in order to better serve this population. Finally, we are building our partnership with the Family Medicine residency program to encourage increased participation by these residents.

## Conclusion

The Well-Woman clinic is a longitudinal partnership between Ob/Gyn residents and the HNHC to provide care to refugee women while fostering resident advocacy, collaboration, and longitudinal engagement in local global health. Integrating this partnership into the formal curriculum will promote sustainability and universal exposure for residents.
